# Segmentary ureteral resection followed by ureteroneocystostomy associated with radical hysterectomy and partial cystectomy in a patient with bulky residual disease after chemoirradiation for invasive cervical cancer - A case report -

**Published:** 2014

**Authors:** N Bacalbaşa, I Bălescu

**Affiliations:** * ”Carol Davila” University of Medicine and Pharmacy, Bucharest, Romania; ** ”Ponderas” Hospital, Bucharest, Romania

**Keywords:** locally advanced cervical cancer, ureteral resection and reimplantation

## Abstract

Cervical cancer represents the second most frequent malignancy in women worldwide, a significant number of cases still being diagnosed in an advanced stage of the disease. In some of these cases, local invasion is already present at the moment of diagnosis and even if neo-adjuvant chemoirradiation is performed in some patients, it persists at the moment of surgery. In these cases, more aggressive surgical procedures are needed in order to obtain a good control of the disease. The case of a 50-year-old patient diagnosed with locally advanced cervical cancer invading the right ureter is presented, in whom this aspect was present even after neo-adjuvant chemoirradiation and in whom a total radical hysterectomy with bilateral en bloc adnexectomy with partial cystectomy and the invaded zone of the right ureter was performed. The ureter was then mobilized and reimplanted in the urinary bladder through a neocystostomy. The postoperative course was uneventful.

## Introduction

Despite the introduction of largely applied screening tests for the early detection of cervical cancer, this malignancy still represents an important problem of health for women worldwide, being responsible for almost 273000 deaths annually [**1**,**2**]. Standard treatment in cases presenting large cervical tumors with local invasion consists in neoadjuvant chemo-irradiation and brachytherapy followed by surgery [**3**]. However, not in all cases, the neo-adjuvant treatment can provide a significant diminish of the tumoral invasion, the bulky disease still being present at the moment of surgery. In these cases, more aggressive surgical procedures are needed. Although pelvic exenteration remains the cornerstone in the locally invasive cervical cancer, there are cases in which invasion is not as extended, so more conservative procedures might be tempting. We present the case of a 50-year-old patient diagnosed with locally invasive cervical cancer involving the right ureter in whom a total radical hysterectomy with bilateral en bloc adnexectomy with partial cystectomy and partial resection of the right ureter with ureteral reimplantation in the urinary bladder was performed.

**Case report**

**Fig. 1:**
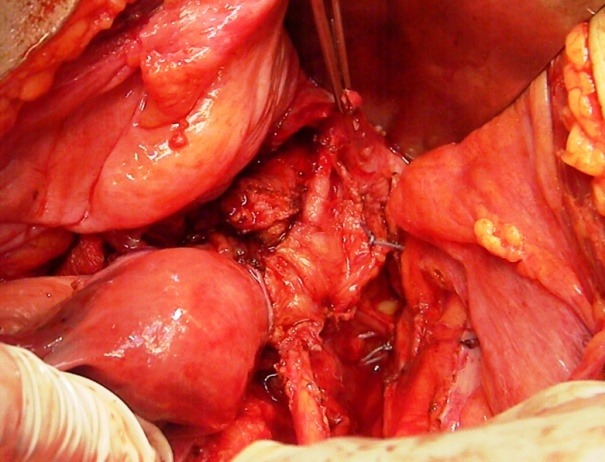
The right ureter entering the cervical tumor

**Fig. 2:**
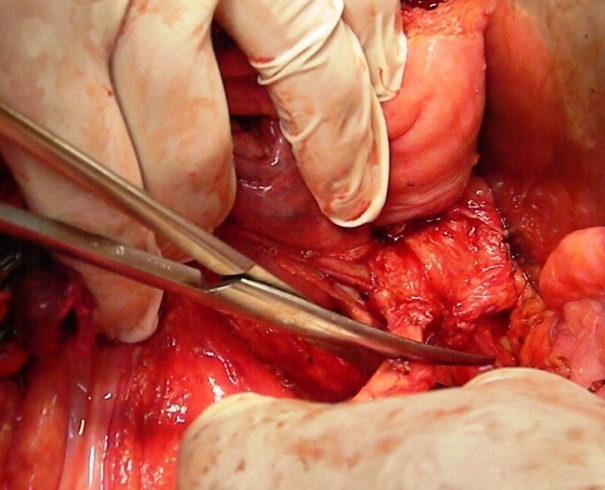
Sectioning the right ureter

**Fig. 3:**
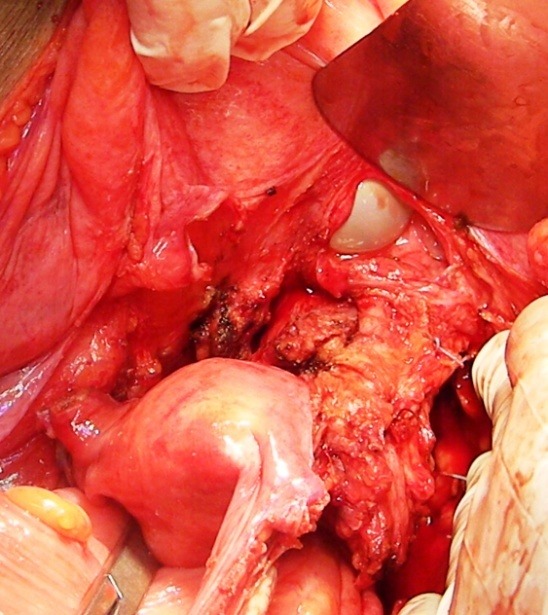
Resection of the invaded zone of the urinary bladder

**Fig. 4:**
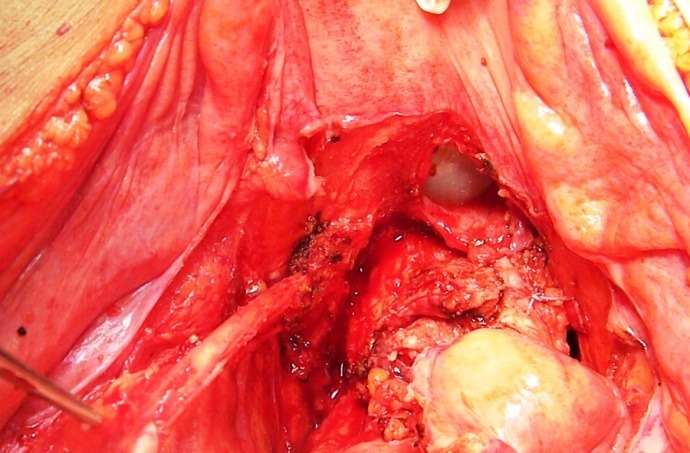
The left ureter entering the urinary bladder, free of any tumoral invasion

**Fig. 5:**
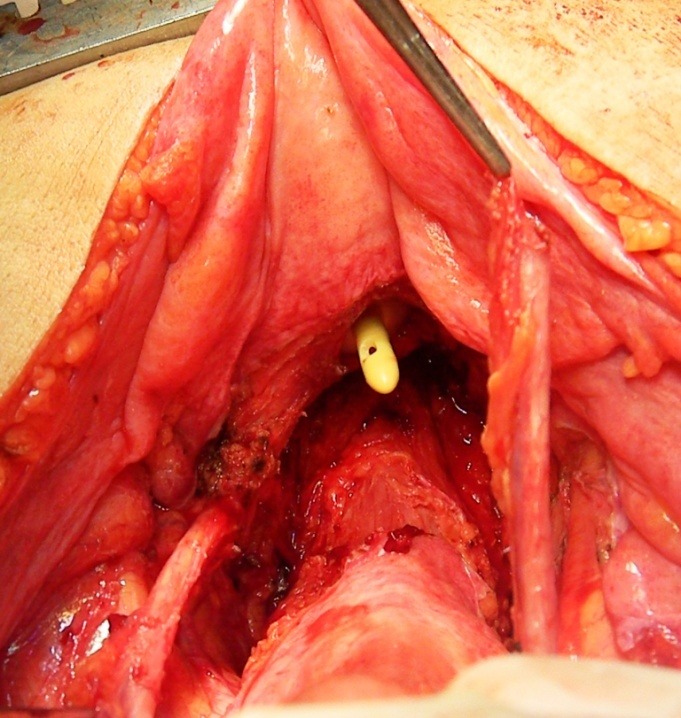
The final aspect after total radical hysterectomy with bilateral en bloc adnexectomy with partial cystectomy and resection of the right distal ureter

**Fig. 6:**
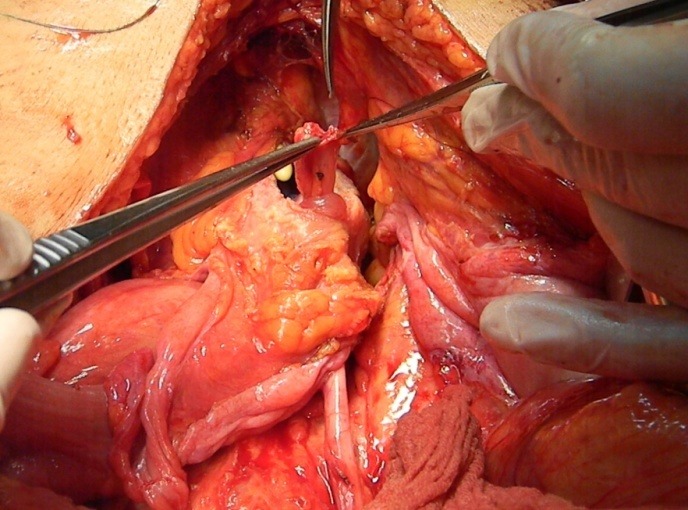
Reimplantation of the right ureter through uretero-neocystostomy

**Fig. 7:**
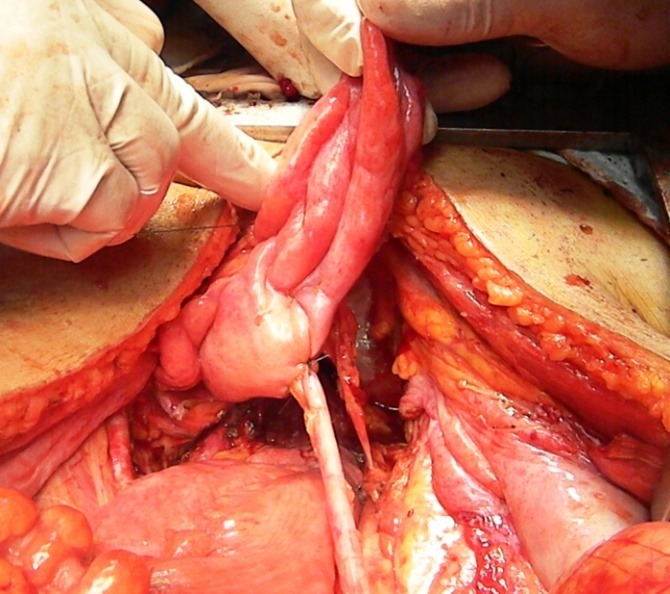
The final aspect after reimplantation. Preservation of the superior vesical artery as a vascularisation source for the mobilized urinary bladder

**Fig. 8:**
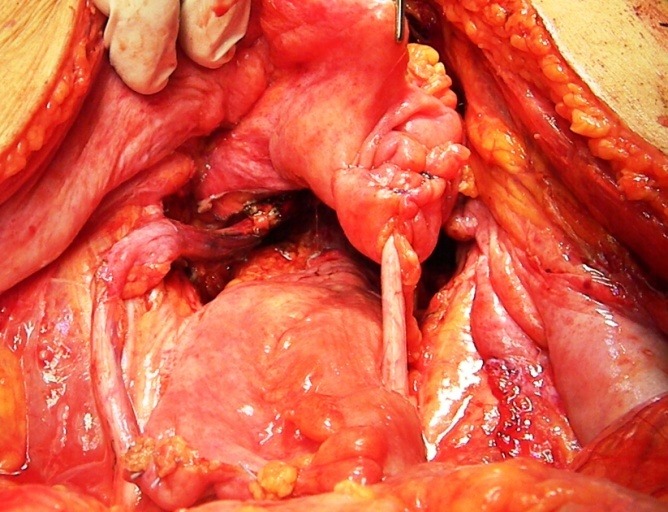
The final aspect after right ureter reimplantation

**Fig. 9:**
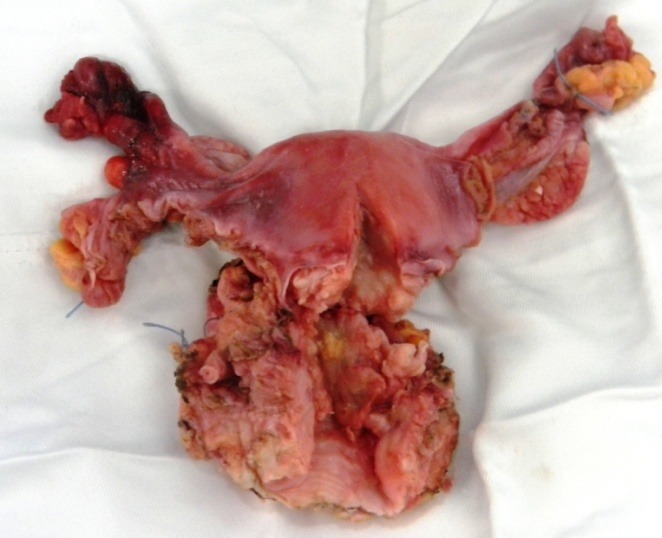
The specimen: total hysterectomy with bilateral en bloc adnexectomy with partial cystectomy and distal ureterectomy

The 50-year-old patient presented to our service for massive vaginal bleeding associated with pelvic pain and hematuria. The local exam revealed a cervical tumor extended to the posterior wall of the urinary bladder and the biopsy confirmed the presence of a well-differentiated squamous cell carcinoma, so the patient was addressed for neo-adjuvant chemoirradiation. One month after finishing the standard treatment of chemotherapy associated with brachytherapy she was readdressed to the surgery department. Preoperative examinations revealed the persistence of a bulky cervical tumor of 4 cm, invading the right distal ureter with secondary hydronephrosis. A total radical hysterectomy with bilateral en bloc adnexectomy with minimal cystectomy and segmentary resection of the invaded zone of the distal right ureter was performed (**Fig. 1**-**5**). A complete mobilization of the remnant ureter was performed while associated with a partial mobilization of the urinary bladder. The ureter was re-implanted through a neocystostomy (**Fig. 6**-**8**). The uretero-neocystostomy was done through a transverse cystostomy by suturing the spatulated end of the ureter directly with separable 4-0 stitches and the anastomosis was protected by introducing a double J urinary catheter which was removed through cystoscopy 1 month later. The patient was discharged in the 10th postoperative day free of any complication. The histopathological exam of the specimen (**Fig. 9**) confirmed the results of the preoperative biopsies.

## Discussions

Studies have shown that the local tumor spread is not random, being conducted by the existence of compartmental borders which represent in fact natural barriers for neoplastic dissemination [**4**]. In pelvis, these compartmental borders are represented by the transversal planes, which separate the organs having different embryologic origins [**5**]. In pelvis, these barriers are represented by the posterior wall of the urinary bladder, the anterior wall of the rectum and the peritoneal reflections between these organs. These plans have the capacity to limit the extent of a cervical tumor but once the neoplastic process continues growing, these borders will be destroyed and the surrounding organs will be invaded [**4**,**5**]. In these cases, more aggressive surgical procedures are needed in order to obtain a good local control of the tumor. Since Brunschwig reported it for the first time in the early 40’s, pelvic exenteration became the cornerstone in treating these patients [**4**-**9**]. This kind of ultra-radical surgery refers to the *en bloc resection* of all the invaded organs [**10**].

Once the standard protocol for cervical cancer included the association of neoadjuvant chemo-irradiation, in the last few decades, the incidence of the large local invasive cervical tumors decreased [**11**].

Anyway, the persistence of bulky cervical tumors after neo-adjuvant therapy represents a poor prognostic factor [**12**,**13**], only a limited percent of these cases having the opportunity to benefit from curative surgery. In their study, Houvenaeghel et al. [**11**] demonstrated that only 63% of the patients presenting bulky tumors (>2 cm after neo-adjuvant therapy) could benefit from curative surgery, while this percent reached up to 97,3% when it came about cases in which the tumor diminished under oncologic treatment. The influence of curative surgery was also reflected in terms of overall survival when the two groups were compared: 3 years and 5 years survival reported rates were of 42,7% and 36,6% respectively for patients with bulky disease, 64,9% and 55,6% respectively for cases with tumors <2 cm after neoadjuvant chemotherapy and 0% for patients in whom curative surgery was not possible. The results of this study sustain the idea that surgery is the only potential curative treatment even in cases with poor responses to neo-adjuvant chemo-irradiation. This way, surgery can provide a good local control of the tumor and, subsequently, may impede the apparition of local recurrences, which are known to be as the most important pattern of failure in patients with advanced cervical cancer [**14**].

However, in the cases presenting the local invasion even after neo-adjuvant therapy, pelvic exenteration is not needed all the time. In selected cases in which the local invasion is not so extended, more conservative procedures can be tempting. In these cases, segmental resections of the ureter or partial cystectomies may be enough [**15**,**16**]. In our case, the local invasion was confined to a single ureteral ostium, while the left ureter was free of any tumoral invasion; this fact giving us the possibility of preserving almost entirely the urinary bladder. A partial en bloc cystectomy with the right ureteral ostium was performed, the defect was sutured and the right ureter was reimplanted through neo-cystostomy and the anastomosis was protected by inserting a double J urinary catheter which was removed three weeks after through cystoscopy.

This method of reconstruction is usually utilized in gynecology in ureteral re-implantation after iatrogenic injuries within 5 cm distally of the pelvic ureter but it was successfully implemented in cases presenting locally advanced malignancies extended to the distal ureter [**17**-**20**].

In their study conducted in a Gynecology service, Hoffman et al. reported 46 cases who needed ureteral surgery between 1997 and 2004. Ureteral resection for gynecologic malignancies was performed in 16 cases; all of them underwent urinary tract reconstruction consisting in uretero-neocystostomy. They reported the association of a partial cystectomy in a single case. The postoperative long term follow up identified one patient who died of recurrent disease 6 months after surgery and another one who developed a recurrent tumor 14 months after surgery [**21**].

Harada et al. reported the utilization of ureteral reimplantation as a method of reconstruction after segmentary ureteral resections in locally invasive cervical cancer in 2 patients with good functional results [**22**].

## Conclusions

The persistence of the bulky cervical tumor after neo-adjuvant chemo-irradiation usually shows the presence of an aggressive malignancy; in these cases, extended surgical resections seem to be a potential curative option in order to obtain a good control of the disease. In cases presenting a limited extent of the malignant process, more conservative procedures can be tempting, with good oncologic outcomes. Although ureteral reimplantation through neocystostomy is usually performed for iatrogenic injuries of the distal ureter, studies have shown that it is perfectly feasible in gynaecologic oncology too, especially if the invaded zone is not too large and the two anastomotic partners – the remnant ureter and the urinary bladder can be mobilized enough, and, in the same time, efficient vascularisation sources can be preserved.
